# DDRGK1 Regulates NF-κB Activity by Modulating IκBα Stability

**DOI:** 10.1371/journal.pone.0064231

**Published:** 2013-05-10

**Authors:** Peng Xi, Deqiang Ding, Junzhi Zhou, Miao Wang, Yu-Sheng Cong

**Affiliations:** 1 Key Laboratory for Cell Proliferation and Regulation Biology of Ministry of Education, Institute of Cell Biology, College of Life Sciences, Beijing Normal University, Beijing, China; 2 Institute of Aging Research, Hangzhou Normal University School of Medicine, Hangzhou, China; Kyushu Institute of Technology, Japan

## Abstract

NF-κB is a ubiquitously expressed transcription factor that regulates a large number of genes in response to diverse physiological and pathological stimuli. The regulation of the transcriptional activity of NF-κB is often dependent on its interaction with IκBα. Proteins that bind to IκBα are critical regulators of NF-κB activity. DDRGK1 is a member of the DDRGK domain-containing protein family with unknown function. In this study, we showed that the depletion of DDRGK1 inhibits cell proliferation and invasion. Microarray analysis indicated that the expression of NF-κB target genes showed the most significant decrease after depleting of DDRGK1, suggesting that DDRGK1 may play an important role in the NF-κB signaling pathway. We further demonstrated that DDRGK1 interacts with IκBα and regulates its stability, thereby regulates the NF-κB transcriptional activity. Our findings identify DDRGK1 as an important regulator of the NF-κB pathway.

## Introduction

The nuclear factor-κB (NF-κB) family is a group of inducible transcription factors that is ubiquitously expressed in almost all cell types and regulates the expression of a large number of genes in response to physiological and pathological stimuli and other stress responses that require the rapid reprogramming of gene expression. NF-κB plays a fundamental role in not only inflammatory and immune responses but also tumorigenesis [1], [2], [3].

This family of transcription factors consists of five members, p65 (RelA), RelB, c-Rel, p50/p105 (NF-κB1), and p52/p100 (NF-κB2). In unstimulated cells, NF-κB family proteins exist as homo- or heterodimers that are bound to inhibitor IκB family proteins; the IκB proteins mask the nuclear localization signals (NLSs) of the NF-κB proteins and thereby ensure that these proteins remain sequestered in an inactive state in the cytoplasm [1], [2]. Upon its activation by a variety of extracellular stimuli, the IκB protein undergoes rapid phosphorylation, ubiquitination, and ultimately proteolytic degradation; this process allows NF-κB to translocate to the nucleus and activate the expression of specific genes [4]. The p65/p50 heterodimer is the first form of NF-κB to be identified, and it is predominantly regulated by IκB. IκBα masks the NLS of p65 without blocking the NLS of p50; thus, the p65 subunit of NF-κB provides the gene regulatory function of the heterodimer. The dynamic balance between the cytosolic and nuclear pools of the p65/p50 heterodimer changes in response to a variety of stimuli, producing physiological and pathological responses [1].

The human DDRGK domain-containing 1 (DDRGK1) gene (also known as C20orf116, CT116 and Dashurin) is located on chromosome 20p13 and has an unknown function [5]. DDRGK1 has been highly conserved during evolution, suggesting that it may exert fundamental cellular functions. DDRGK1 has no characteristic features of functional domains or motifs, except for a conserved PCI domain (Proteasome, COP9, and initiation factor-3) at the C-terminus. This domain is known as a protein-protein interaction motif [5], [6], [7].

It has been shown that DDRGK1 is located in the endoplasmic reticulum (ER) and its expression is induced by ER stress [8]. Recent studies indicate that DDRGK1 interacts with a protein complex containing UFL1 (UFM1-specific ligase 1), the putative tumor suppressor LZAP/C53 and UFM1 (ubiquitin fold modifier 1), and both UFL1 and LZAP/C53 have a regulatory role in the NF-κB pathway [8], [9], [10]. However, the function of this protein complex and its regulatory mechanisms in the NF-κB pathway remain largely unknown. In particular, the role of DDRGK1 in NF-κB pathway has not previously been investigated. In this study, we identified DDRGK1 as an important regulator of the NF-κB pathway. We demonstrated that DDRGK1 interacts with IκBα and regulates its stability, thereby regulates the transcriptional activity of NF-κB and the expression of NF-κB target genes.

## Results

### The Depletion of DDRGK1 Expression Inhibits Cell Proliferation

**Figure 1:**
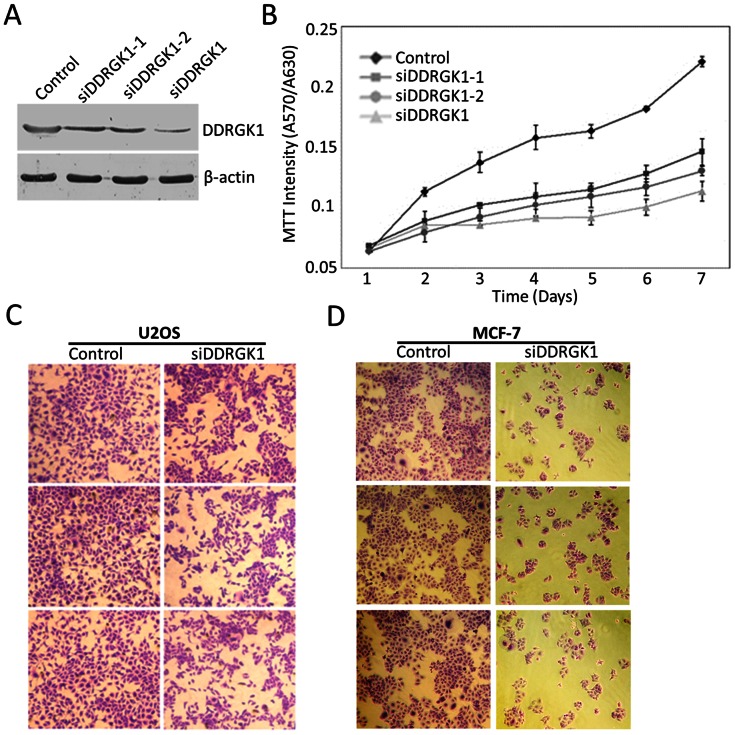
The depletion of DDRGK1 expression inhibits cell proliferation. (A) Western blot analysis of the downregulation of DDRGK1 by DDRGK1 siRNA1, siRNA2, or a mixture of siRNA1 and siRNA2. U2OS cells were transfected with two specific individual or mixed DDRGK1 siRNAs (siDDRGK1). The depletion of DDRGK1 expression was confirmed by immunoblotting. (B) The depletion of DDRGK1 expression inhibits cell proliferation in U2OS cells. U2OS cells were transfected with individual or mixed DDRGK1 siRNAs, and growth curves were generated at the indicated times. The data presented were from three independent experiments. (C) U2OS cells were transfected with mixed DDRGK1 siRNAs or a control siRNA. 72 hours after transfection, cells were stained with Giemsa solution. (D) The depletion of DDRGK1 expression inhibits cell proliferation in MCF-7 cells. MCF-7 cells were transfected with mixed DDRGK1 siRNAs or a control siRNA. 72 hours after transfection, cells were stained with Giemsa solution.

Human DDRGK1 is an abundant cytoplasmic protein [8], [9]. To investigate the cellular function of the DDRGK1 protein, we examined the effect of DDRGK1 depletion by the two specific siRNAs in U2OS cells (Figure 1A). The proliferation of U2OS cells transfected with either of the two individual DDRGK1 siRNAs or the siRNA mixture was assessed by MTT assay and Giemsa staining. The resulting data indicate that the depletion of DDRGK1 expression significantly inhibits cellular proliferation in U2OS cells (Figure 1, B and C). Similarly, we found that the depletion of DDRGK1 inhibits proliferation in MCF-7 cells (Figure 1D). Together, these results indicate that DDRGK1 is involved in the cell proliferation.

### The Depletion of DDRGK1 Inhibits the Expression of Cyclin D1

**Figure 2:**
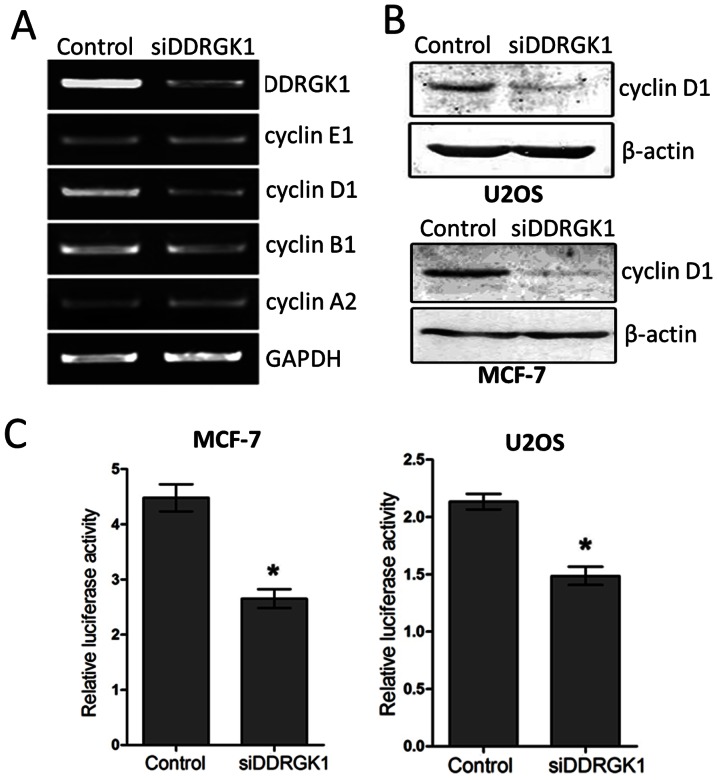
DDRGK1 regulates the expression of cyclin D1. (A) The depletion of DDRGK1 inhibits the expression of cyclin D1 in MCF-7 cells. MCF-7 cells were transfected with mixed DDRGK1 siRNAs or a control siRNA. The expression of DDRGK1 and four cyclin genes were analyzed by RT-PCR. GAPDH was used as a loading control. (B) The depletion of DDRGK1 decreases the expression of cyclin D1. MCF-7 cells and U2OS cells were transfected with mixed DDRGK1 siRNAs or a control siRNA. The level of cyclin D1 protein was determined by immunoblotting. (C) The depletion of DDRGK1 decreases the cyclin D1 promoter activity. U2OS cells and MCF-7 cells were transfected with mixed DDRGK1 siRNAs or a control siRNA along with the cyclin D1 promoter reporters. Relative luciferase activity was determined by the dual luciferase assay system. In this analysis and subsequent luciferase assays, fireﬂy luciferase activity was normalized with the Renilla luciferase reporter of pRL-TK. The data are expressed as the mean±standard error from three independent experiments.

To further explore the effect of DDRGK1 on cell proliferation, we examined the expression of cyclins. We observed that the depletion of DDRGK1 decreased the mRNA expression of cyclin D1, while had no effects on the expression of cyclin A2, cyclin B1, cyclin E1 in MCF-7 (Figure 2A). We further confirmed that the cyclin D1 protein levels were decreased in both U2OS and MCF-7 cells transfected with DDRGK1 siRNA_S_ (Figure 2B). Using luciferase reporter assays with the promoter of cyclin D1, we found that the depletion of DDRGK1 by siRNAs significantly inhibited the cyclin D1 promoter activity in both U2OS and MCF-7 cells (Figure 2C). Taken together, these results indicate that DDRGK1 regulates the expression of cyclin D1 at transcriptional levels.

### The Depletion of DDRGK1 Inhibits Cell Migration and Invasion in U2OS Cells

**Figure 3:**
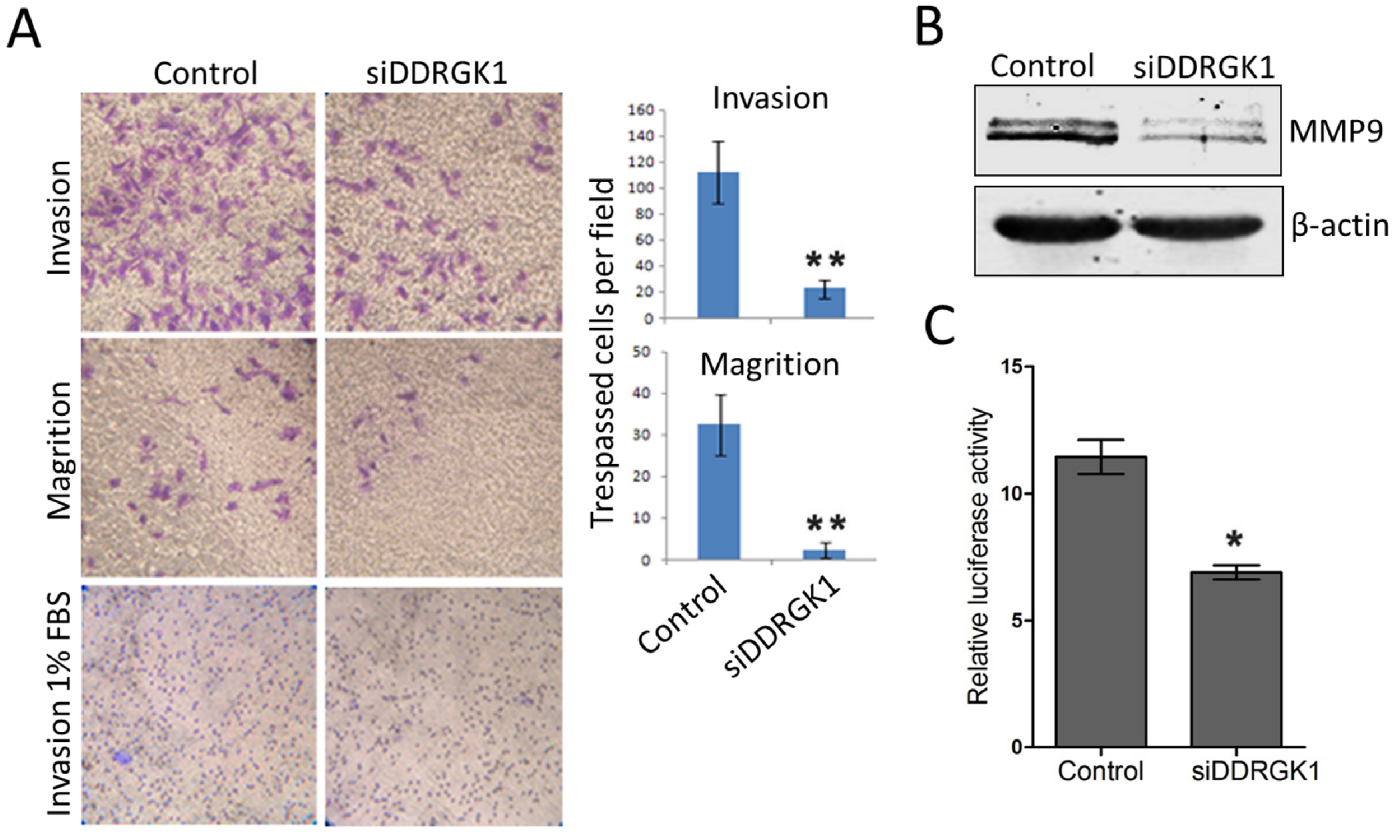
The depletion of DDRGK1 inhibits cell migration and invasion in U2OS cells. (A) U2OS cells were transfected with mixed DDRGK1 siRNAs or a control siRNA. Cell migration and invasion through transwells with or without Matrigel were analyzed by the direct counting of the migrated cells. Medium containing 1% FBS in the lower chamber served as a control. (B) The depletion of DDRGK1 decreases the expression of MMP9. U2OS cells were transfected with mixed DDRGK1 siRNAs or a control siRNA. The level of MMP9 protein expression was determined by immunoblotting. (C) The depletion of DDRGK1 decreases the MMP9 promoter activity. U2OS cells were transfected with mixed DDRGK1 siRNAs or a control siRNA along with the MMP9 promoter reporters. Relative luciferase activity was determined by the dual luciferase assay system as described in Figure 2C.

We then examined the effects of DDRGK1 on cell migration and invasion through a Matrigel barrier. We observed that the depletion of DDRGK1 by the siRNA mixture resulted in an approximately 5-fold decrease in cellular migration ability and an even more pronounced reduction in cell invasion (Figure 3A), suggesting a role for DDRGK1 in cell migration and invasion. Matrix metalloproteinases 9 (MMP9), known as gelatinase B, belongs to a family of secreted or membrane-associated zinc-dependent extracellular endopeptidases that enhance invasion and metastases [11]. To determine whether DDRGK1 regulates the expression of MMP9, we examined the expression of MMP9 in DDRGK1-depleted U2OS cells. The results show that the depletion of DDRGK1 significantly decreased the MMP9 expression (Figure 3B). Furthermore, the depletion of DDRGK1 by siRNAs significantly inhibits MMP9 promoter activity in U2OS cells (Figure 3C), indicating that DDRGK1 regulates the expression of MMP9 at transcriptional levels.

### The Depletion of DDRGK1 Inhibits the Expression of NF-κB Target Genes in U2OS Cells

**Figure 4:**
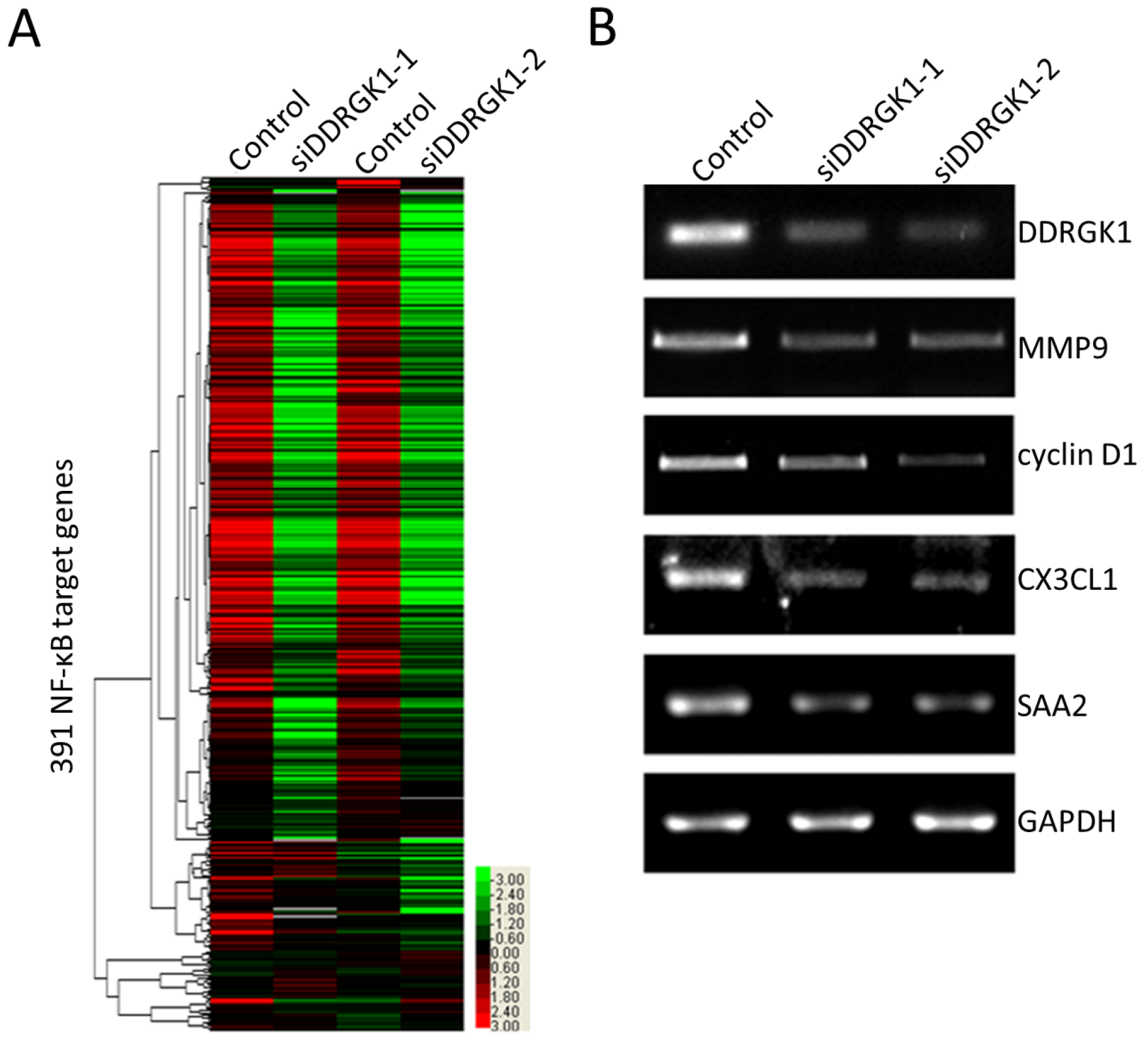
The depletion of DDRGK1 inhibits the expression of NF-κB target genes in U2OS cells. (A) The expression levels of NF-κB target genes were analyzed by microarray. U2OS cells were transfected with DDRGK1 siRNA1 and siRNA2, respectively. The mRNAs were isolated for microarray analysis. The ratio of the abundance of transcripts of each gene in a given sample to its median abundance is represented by the color of the corresponding cell in the Tree View-generated diagram. Green cells represent the genes with transcript levels below their median abundances, black cells represent genes with transcript levels equal to their median abundances, and red cells represent genes with transcript levels that are greater than their median abundances. Gray cells indicate technically inadequate or missing data. The color saturation of each cell reflects the magnitude of the ratio relative to the mean for each gene. NF-κB target genes were selected based on information from the Boston University network (http://www.bu.edu/nf-kb/gene-resources/target-genes/). (B) RT-PCR analysis of selected NF-κB target genes was performed to validate the results of the microarray. U2OS cells were transfected with DDRGK1 siRNA1 or siRNA2, and total RNAs were extracted. The expression of DDRGK1 and four selected NF-κB target genes (MMP9, cyclin D1, CX3CL1 and SAA2) were analyzed by RT-PCR with the specific primers listed in the Experimental Procedures. GAPDH was used as a loading control.

To gain further insights into the role of DDRGK1 in cell migration and invasion, we performed a “35K Human Genome Array” oligonucleotide microarray (see “Materials and methods”). The results of this analysis indicated that of the examined genes, NF-κB target genes demonstrated the most significant decreases in expression after the depletion of DDRGK1. The hierarchical clustering of 391 NF-κB target genes (http://www.bu.edu/nf-kb/gene-resources/target-genes/) revealed systematic features of gene expression that were affected by the transfection of the DDRGK1 siRNAs compared with the control siRNA (Figure 4A). Among the 391 NF-κB target genes, 138 were downregulated by transfection with DDRGK1 siRNA1, and 131 were downregulated by transfection with DDRGK1 siRNA2, compared to control siRNA transfection. The NF-κB target genes that were consistently down-regulated by more than two-fold after transfection with both DDRGK1 siRNA1 and DDRGK1 siRNA2 are listed in Table S1. To confirm the results of the microarray analysis, the expression levels of 4 selected NF-κB target genes, including MMP9, cyclin D1, CX3CL1, and SAA2, were analyzed by RT-PCR. Consistent with the microarray data, the expression of these genes was significantly reduced by the siRNA-mediated down-regulation of DDRGK1 (Figure 4B). In combination, these results indicate that DDRGK1 regulates the expression of NF-κB target genes at transcriptional levels.

### DDRGK1 Regulates the NF-κB Transcriptional Activity

**Figure 5:**
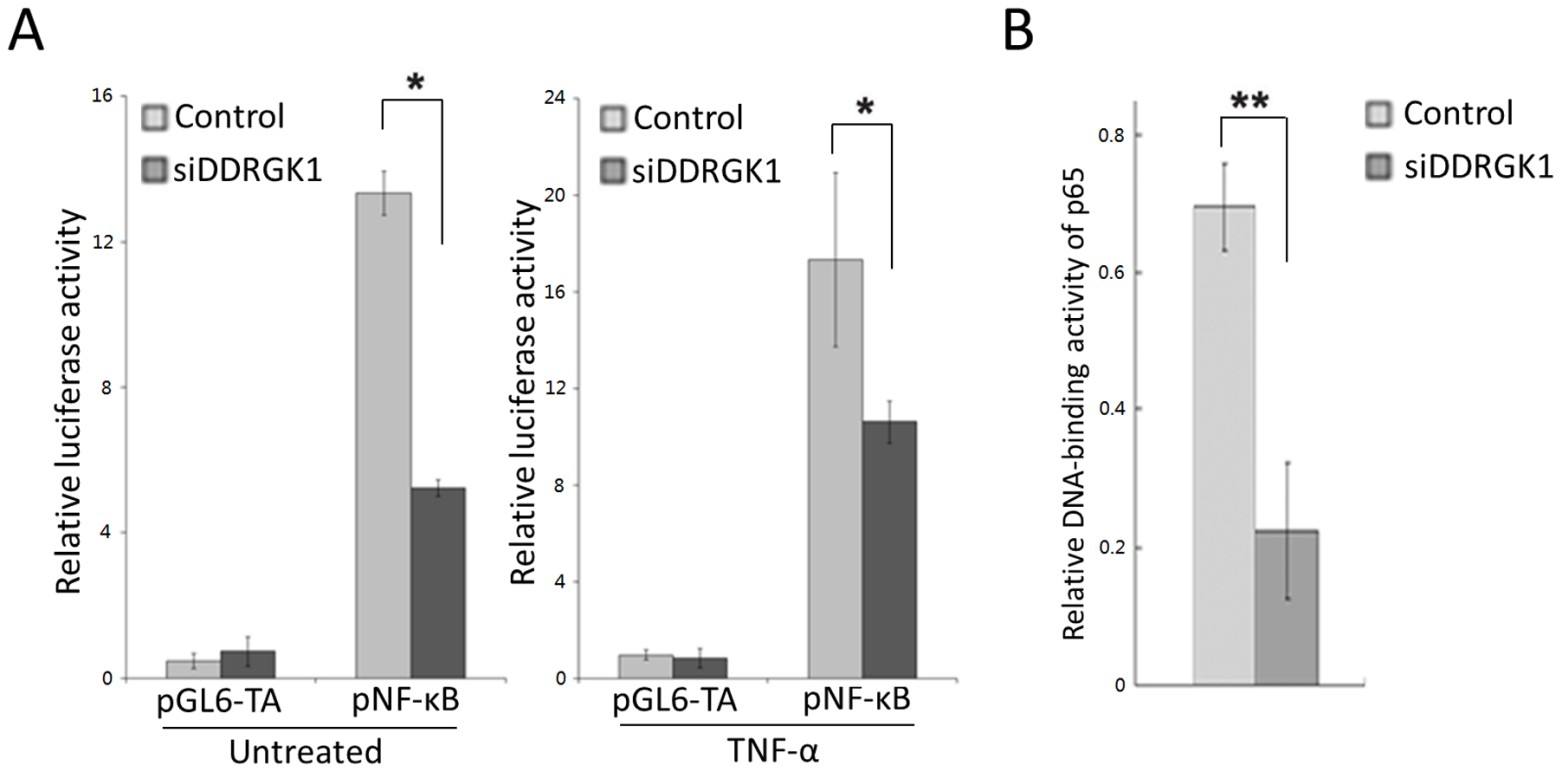
The depletion of DDRGK1 expression inhibits NF-κB transcriptional activity. (A) The depletion of DDRGK1 decreases NF-κB activity. U2OS cells were transfected with either mixed DDRGK1 siRNAs or a control siRNA, along with the control pGL6-TA or the pNF-κB luciferase reporter. 48 hours after transfection, the cells were treated with 10 ng/ml TNF-α for 4 hours or left untreated as control. Luciferase activities were determined by the dual luciferase assay system. (B) The depletion of DDRGK1 inhibits the DNA-binding activity of p65. U2OS cells were transiently transfected with mixed DDRGK1 siRNAs or a control siRNA, and nuclear proteins were extracted. The DNA-binding activity of p65 toward a consensus κB site was then assessed using the TransAM NF-κB p65 transcription factor assay kit.

To investigate the transcriptional regulation of NF-κB target genes by DDRGK1, NF-κB-dependent luciferase reporter assays were performed in U2OS cells transfected with a reporter construct containing NF-κB binding sites along with either DDRGK1 siRNAs or a control siRNA. Consistent with the microarray data, we found that the depletion of DDRGK1 by siRNA mixture specifically inhibited NF-κB luciferase reporter activity. A similar inhibition of NF-κB luciferase reporter activity was also observed after the stimulation of NF-κB activity by treatment with TNF-α (Figure 5A).

We then measured the DNA-binding activity of NF-κB in cells transfected with DDRGK1 siRNA mixture or the control siRNA. Nuclear extracts were prepared and the DNA-binding activity of p65 toward a consensus κB site was then assayed using the TransAM NF-κB p65 transcription factor assay kit. We found that the depletion of DDRGK1 significantly inhibited the DNA-binding activity of p65/RelA (Figure 5B) in U2OS cells. Collectively, these results demonstrate that DDRGK1 regulates NF-κB transcriptional activity in U2OS cells.

### DDRGK1 Regulates the Stability of IκBα

**Figure 6:**
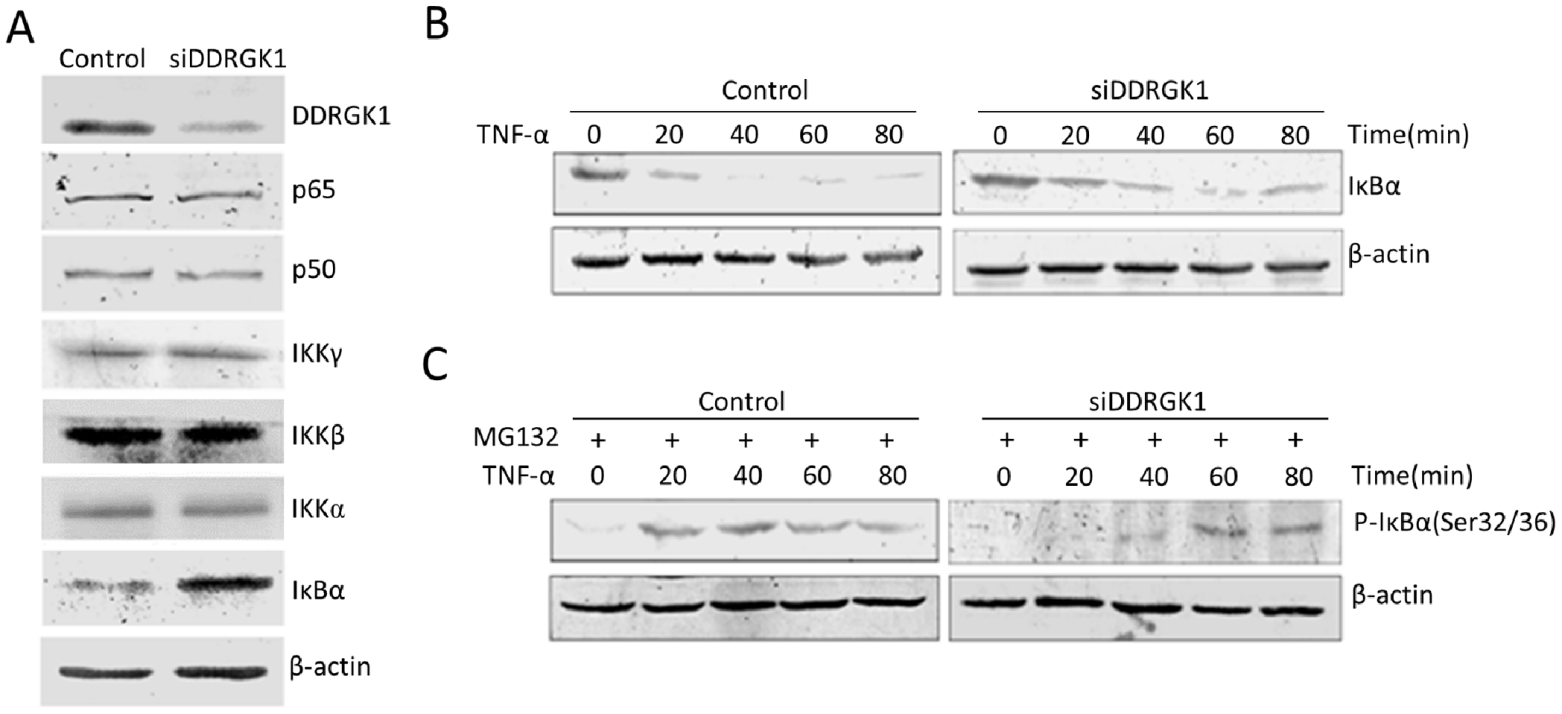
DDRGK1 regulates the stability of IκBα. (A) Effect of down-regulation of DDRGK1 on the NF-κB family protein expression. U2OS cells were transiently transfected with mixed DDRGK1 siRNAs or a control siRNA. 48 hours after transfection, cell lysates were prepared and subjected to immunoblot analysis of NF-κB-related protein expression (p50, p65, IKKα, IKKβ, IKKγ, IκBα). The β-actin was used as a loading control. (B) DDRGK1 regulates the stability of IκBα. U2OS cells were transfected with mixed DDRGK1 siRNAs or a control siRNA. 48 hours after transfection, cells were treated with 10 ng/ml TNF-α at indicated time. The cell lysates were prepared and subjected to immunoblotting analysis of IκBα expression. (C) DDRGK1 regulates the phosphorylation of IκBα. U2OS cells were transfected with mixed DDRGK1 siRNAs or a control siRNA. 48 hours after transfection, cells were treated with 10 ng/ml TNF-α at indicated time in the presence of 20 µM MG132. The cell lysates were subjected to immunoblotting analysis with the phospho-IκBα antibody. β-actin was used as a loading control.

To understand the mechanism of DDRGK1 in the regulation of NF-κB transcriptional activity, we examined the effects of the down-regulation of DDRGK1 on the levels of NF-κB family protein expression. The depletion of DDRGK1 by siRNAs resulted in a notable increase in IκBα expression. However, the expression of other members of the NF-κB family, including p65, p50, and IKKα, IKKβ, IKKγ, appears to be unchanged with the depletion of DDRGK1 (Figure 6A). These results suggest that IκBα may be the target through which DDRGK1 regulates NF-κB.

The degradation of IκBα is a central event in the NF-κB activation process, and it is initiated by the specific phosphorylation of IκBα. Following this phosphorylation, IκBα is ubiquitinated and then degraded by the proteasome, allowing p65/p50 NF-κB heterodimers to translocate into the nucleus and mediate NF-κB activity [1]. To investigate the effects of DDRGK1 on the phosphorylation and degradation dynamics of IκBα, the levels of IκBα expression and phosphorylation were examined every 20 minutes after stimulation with TNF-α in U2OS cells transfected with either control or DDRGK1 siRNAs. As expected, the protein level of IκBα in the control cells decreased quickly and was almost undetectable at 40 minutes after stimulation with TNF-α. However, the degradation of IκBα was significantly delayed, by up to 80 minutes, in DDRGK1-depleted cells (Figure 6B). Correspondingly, the phosphorylation of IκBα was effectively inhibited after stimulation with TNF-α in the presence of the proteasome inhibitor MG132. In the control cells, the phosphorylation of IκBα was readily detectable at 20 minutes of TNF-α treatment. However, in the DDRGK1-depleted cells, the phosphorylation of IκBα was not observed until 60 minutes of TNF-α treatment (Figure 6C). These results indicate that DDRGK1 regulates NF-κB activity by interfering with IκBα phosphorylation and degradation.

### DDRGK1 Interacts with IκBα

**Figure 7:**
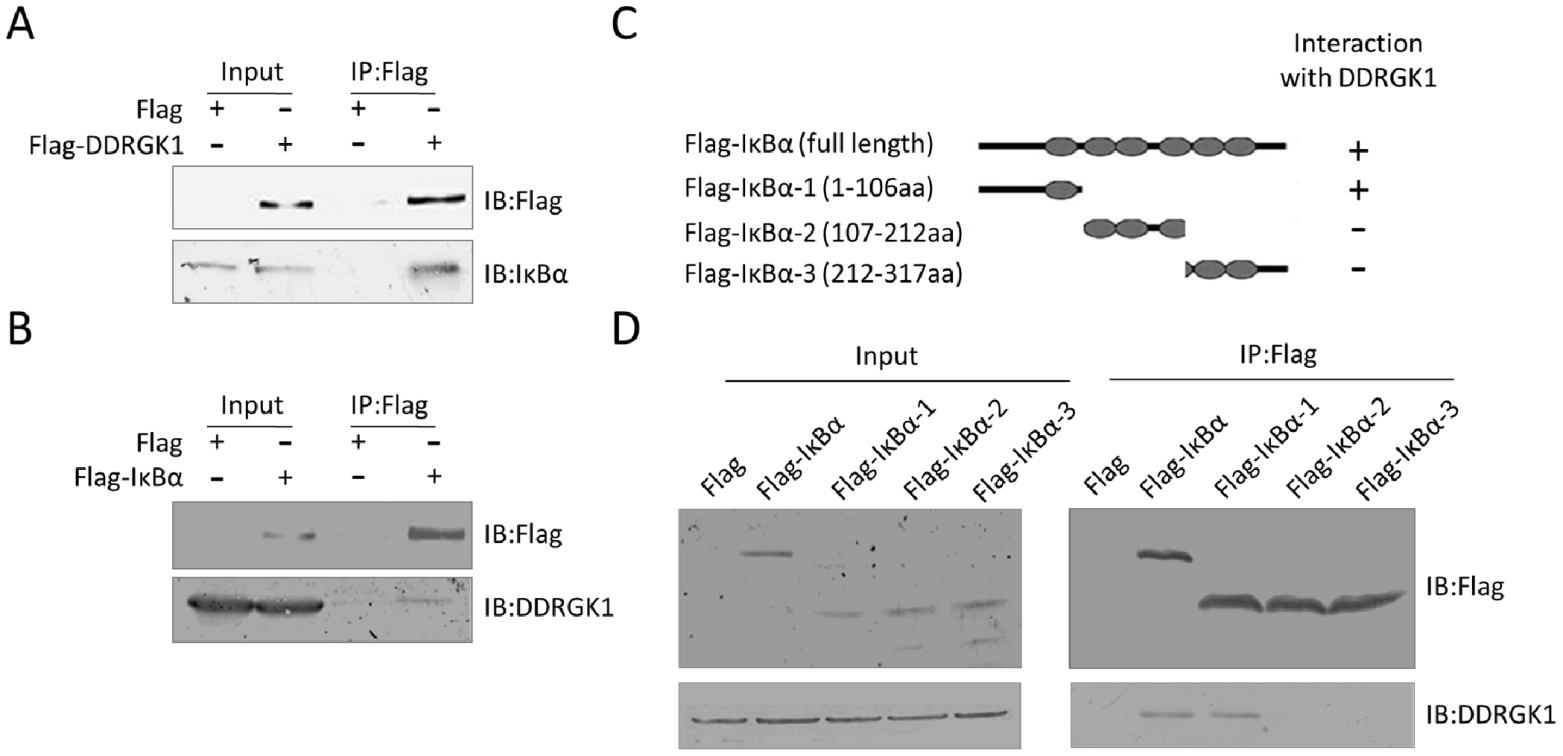
DDRGK1 interacts with IκBα. (A) Immunoprecipitation (IP) analysis of the interaction of Flag-tagged DDRGK1 with endogenous IκBα. 293T cells were transfected Flag-tagged DDRGK1 construct or a control vector. Cell lysates were immunoprecipitated with anti-Flag resin and then analyzed by immunoblotting with IκBα and Flag antibodies. 5% of each cell lysate was loaded as input. (B) Immunoprecipitation (IP) analysis of the interaction of Flag-tagged IκBα with endogenous DDRGK1. 293T cells were transfected Flag-tagged IκBα construct or control vector. Cell lysates were immunoprecipitated with anti-Flag resin and then analyzed by immunoblotting with DDRGK1 and Flag antibodies. 5% of each cell lysate was loaded as input. (C) Diagram of full-length (Flag-IκBα) and three truncation mutant constructs (Flag-IκBα-1, Flag-IκBα-2, Flag-IκBα-3). (D) The N-terminal portion of IκBα is sufficient for its interaction with DDRGK1. Flag-tagged full-length or three truncation mutant constructs of IκBα were transiently transfected into 293T cells. The Flag-tagged full-length and mutant IκBα were immunoprecipitated with anti-FLAG resin and then immunoblotted with Flag and DDRGK1 antibodies.

The shuttling of NF-κB between the nucleus and cytoplasm is tightly regulated by IκBα. To understand how DDRGK1 affects the stability of IκBα, we performed co-immunoprecipitation experiments to examine the interaction of DDRGK1 with IκBα. 293T cells were transfected with either the plasmids expressing Flag-tagged IκBα or empty vectors as a control. The cell lysates were immunoprecipitated using the anti-Flag antibody and subsequently analyzed by immunoblotting with anti-DDRGK1 antibodies. The Flag-tagged IκBα was found to interact with the endogenous DDRGK1 (Figure 7A). Similarly, we readily detected endogenous IκBα in Flag antibody immunoprecipitates from 293T cells transfected with Flag-tagged DDRGK1 expression constructs (Figure 7B), indicating that these two proteins interact with each other in cells.

To define the region of the IκBα protein that is required for DDRGK1 interaction, immunoprecipitation experiments using full-length and three truncation mutants of Flag-tagged IκBα were performed (Figure 7C). Our data indicated that the amino terminus (1–106 aa) of IκBα, which contains critical phosphorylation sites (Ser32 and Ser36) and ubiquitination sites (Lys21 and Lys22) [1], [2], [12], was required for binding to DDRGK1. Neither the carboxyl terminus (213–317 aa) nor the middle portion (107–212 aa) of IκBα bound to DDRGK1 (Figure 7D). Taken together, these data indicate that the amino terminus (1–106 aa) of IκBα interacts with DDRGK1 in 293T cells and this interaction may interfere the IκBα protein stability by blocking its phosphorylation and degradation.

## Discussion

In this report, we identified DDRGK1 as an important regulator of NF-κB. We found that DDRGK1 depletion inhibited cell migration and invasion in U2OS cells. Microarray analysis revealed that the depletion of DDRGK1 by siRNAs specifically inhibited NF-κB target gene expression. We further confirmed that DDRGK1 regulated NF-κB DNA binding activity and the transcriptional activity of an NF-κB luciferase reporter. Importantly, we demonstrated that DDRGK1 interacts with the amino terminus (1–106 aa) of IκBα, which contains critical phosphorylation sites (Ser32 and Ser36) and ubiquitination sites (Lys21 and Lys22), and regulates its phosphorylation and stability, thereby modulating the transcriptional activity of NF-κB and the expression of NF-κB target genes. The NF-κB target genes that are inhibited by DDRGK1 siRNAs include cytokines, chemokines, adhesion molecules and receptors, as well as enzymes that are involved in proliferation, differentiation, apoptosis and stress responses, suggesting an important role for DDRGK1 in the NF-κB signaling pathway.

Translocations in regions near the DDRGK1 gene on chromosome 20p13 have been found to be associated with acute myeloblastic leukemia and acute promyelocytic leukemia [13], [14], and a recent genome-wide association study identified one SNP (rs11697186) located in the DDRGK1 gene that showed strong associations in the minor-allele-dominant model with the decrease in platelet counts that occurs in response to pegylated interferon and ribavirin therapy for chronic hepatitis C [15]. These observations further support the functional relevance of DDRGK1 in NF-κB signaling.

An earlier study demonstrated that C53/LZAP is a putative tumor suppressor that plays important roles in the NF-κB signaling [16]. C53/LZAP directly interacts with p65, inhibits the NF-κB transcriptional activity by regulating the phosphorylation of p65, thereby inhibiting basal and stimulated NF-κB transcriptional activity.

Several recent reports have independently revealed that DDRGK1, C53/LZAP, UFL1 and UFM1 are all components of a large protein complex. Using biochemical and proteomic approaches, Tatsumi et al. demonstrated that DDRGK1 (C20orf116 in their work) is a UFM1-specific substrate and that UFL1 may be the E3 ligase for DDRGK1 ufmylation [17]. In a search for LZAP-interacting proteins, Kwon et al. identified UFL1 (NLBP in their work), DDRGK1 and UFM1 in C53/LZAP immunoprecipitates subjected to proteomic analyses and found that UFL1 has negative effects on the NF-κB pathway [10]. Similarly, Wu et al. demonstrated that C53/LZAP interacts with DDRGK1 and UFL1 (RCAD in their work) and that UFL1 regulates the stability of both C53/LZAP and DDRGK1 [9]. Furthermore, data from this study also suggest that UFL1 may function to stabilize C53/LZAP and block NF-κB signaling [9]. In a recent study, Lemaire et al. have shown that DDRGK1 (UFBP1 in their work), C53/LZAP and UFL1 interact with UFM1 and that these three proteins are components of the UFM1 conjugation pathway [8]. Together, these studies suggest that these four proteins form a novel multi-protein complex. However, the function and regulation of this complex is largely unclear.

DDRGK1 has a highly conserved PCI domain at its carboxyl terminus. The PCI is a protein-protein interaction motif [6]. Proteins that contain this domain appear to be part of large multi-protein complexes. It would be interesting to determine whether the PCI domain in DDRGK1 is an interacting module for the protein complex containing LZAP, UFL1 and UFM1.

Notably, in our immunoprecipitation experiments, we readily detected UFM1-conjugated DDRGK1, LZAP and UFL1, indicating that these four proteins are present in the same complex and are all conjugated by UFM1 (unpublished data). All of these proteins are highly conserved during evolution. Ufm1 is the most recently discovered ubiquitin-like modifier [18]. The function of UFM1 conjugation (ufmylation) is currently unknown. Interestingly, we have observed that UFM1-conjugated proteins, such as DDRGK1, LZAP and UFL1, can easily be detected in UFM1 immunoprecipitates. Thus, ufmylation may not be associated with the proteolysis of these proteins. Whether ufmylation plays a role in the assembly of this multi-protein complex or in the regulation of its activity warrants further investigations.

## Materials and Methods

### Cell Culture, Transfection, Antibodies and Reagents

U2OS, MCF-7, 293T cell lines were from American Type Culture Collection (Manassas, VA, USA) and cultured in Dulbecco’s modified Eagle’s medium (DMEM) with 10% fetal bovine serum (Hyclone), 1% L-glutamine, 100 U/ml penicillin and 100 µg/ml streptomycin at 37°C and 5% CO_2_. The transfection was performed with Lipofectamine 2000 (Invitrogen) according to the manufacturer’s instructions. Antibodies against the following proteins were used for immunoblotting and immunoprecipitation: β-actin, cyclin D1, phospho-NF-κB p65 (Ser536) and phospho-IκBα (Ser32/36) were from Cell Signaling; NF-κB p50, NF-κB p65, IKKα, IKKβ, IKKγ and IκBα were from Abcam; DDRGK1 and Flag were from Sigma. MG132 and TNF-α were purchased from Sigma.

### Plasmid Constructions and siRNAs

The Flag-DDRGK1 and Flag-IκBα expression plasmids were constructed by cloning the full-length DDRGK1 cDNA or the full-length IκBα cDNA into p3xFlag-CMV vectors. The IκBα deletion mutants were obtained from the Flag-IκBα expression plasmid by PCR amplification. These fragments were subsequently cloned into the p3xFlag-CMV vector. Luciferase reporters pNF-κB and pGL6-TA were obtained from Beyotime Institute of Biotechnology. The MMP9 and cyclin D1 promoter reporter vectors were constructed by cloning the MMP9 promoter (−1384 to +1) or the cyclin D1 promoter (−1046 to +180) into the pGL3-Basic vector.

Two previously described DDRGK1-specific siRNAs [9] were used in this study: DDRGK1-1, with a sequence of GAAAAUUGGAGCUAAGAAA, and DDRGK1-2, with a sequence of CCAUAAAUCGCAUCCAGGA.

### Immunoblotting and Immunoprecipitation

Cell lysates were prepared in RIPA buffer. Immunoblotting was performed with specific antibodies. For co-immunoprecipitation, cells were lysed in NP-40 lysis buffer. The extract was incubated with M2 anti-Flag resin (Sigma) overnight at 4°C. The associated proteins were analyzed by immunoblotting.

### Migration and Invasion Assays

Cell migration/invasion assays were performed in 24-well transwells coated with (invasion) or without (migration) 1 mg/ml Matrigel (BD Sciences). U2OS cells were starved overnight in serum-free medium, trypsinized, and washed three times in DMEM containing 1% FBS. The upper chamber was seeded with 2×10^5^ cells in DMEM containing 1% FBS, and 600 µl DMEM containing 10% or 1% FBS was placed in the lower chamber. After 16 h of incubation, the Matrigel and the cells that remained in the upper chamber were removed. The cells on the lower surface of the membrane were fixed in 4% paraformaldehyde and stained with 0.5% crystal violet. The cells in a minimum of six random microscope view fields were counted. All of the experiments were run in duplicate and were repeated three times.

### RT-PCR

Total RNA was extracted from cells using TRIzol reagent (Invitrogen, Paisley, UK) according to the manufacturer’s instructions. Genes of interest were amplified from 2 µg DNase I-treated total RNA using M-MLV Reverse Transcriptase (Promega) and poly dT primer. The primers that were used for PCR were as follows: DDRGK1 (forward: 5′-TGCTGGCTGAGGGGACTATAA-3′; reverse: 5′-CCGCTGTCGGATGAAGTTG-3′), MMP9 (forward: 5′-GGCATTCAGGGAGACGCCCATTT-3′; reverse: 5′-CGAAGAGCTTGTCCCGGTCGTAGTT-3′), cyclin D1 (forward: 5′-CACACGGACTACAGGGGAGT-3′; reverse: 5′-AGGAAGCGGTCCAGGTAGTT-3′), cyclin A2 (forward: 5′-CCAAGAGGACCAGGAGAATA-3′; reverse: 5′-TCCAAGGAGGAACGGTGACA-3′), cyclin B1 (forward: 5′-CGGGAAGTCACTGGAAACAT-3′; reverse: 5′-CCGACCCAGACCAAAGTTTA-3′), cyclin E1 (forward: 5′-AAGTGGATGGTTCCATTTGC-3′; reverse: 5′-TTTGATGCCATCCACAGAAA-3′), CX3CL1 (forward: 5′-GCATGAGGCTAGTGTGGTGT-3′; reverse: 5′-AATGAGTGTTGTCAGGGGGA-3′), SAA2 (forward: 5′-CAGCTCAGCTACAGCACAGATCA-3′); reverse: 5′-CCCGAGCATGGAAGTATTTGTC-3′), GAPDH (forward: 5′-CGGAGTCAACGGATTTGGTCGTAT-3′; reverse: 5′-TGCTAAGCAGTTGGTGGTGCAGGA-3′). The experiments were repeated at least three times. GAPDH was used as an internal control.

### MTT Assay

Cell growth was measured by an MTT [3-(4,5-diethylthiazoly-2-yl)-2,5-diphenyltetrazolium bromide] assay. Briefly, cells were seeded at 500 cells per well in 96-well culture plates in quadruplicate. At various time points, the medium was removed, and the cells were incubated in 180 µl of DMEM containing 5% fetal calf serum and 0.25 mg/ml of MTT (Sigma) at 37°C for 4 h, followed by solubilization with 20% sodium dodecyl sulfate and 50% dimethyl formamide either for another 4 h or overnight at 37°C. The absorbance of each well was measured with a microplate reader at the dual wavelengths of 595 and 655 nm. The viable cell number is proportional to the absorbance.

### cDNA Microarrays and Statistical Analyses

Human genome 70-mer oligonucleotide microarray analyses were performed by the CapitalBio Corporation (Beijing, China). A complete human genome oligonucleotide set from Operon (version 4.0) containing 35,035 oligonucleotide probes representing approximately 25,100 genes and 39,600 transcripts, excluding control oligos, was used. Briefly, mRNAs extracted from U2OS cells that had been transfected with control siRNA were used as a reference control sample to prepare cDNAs labeled with Cy3-dCTP, and mRNAs from U2OS cells transfected with DDRGK1 siRNA were used to prepare cDNAs labeled with Cy5-dCTP. The two differentially labeled cDNA probes were mixed and then simultaneously hybridized to the human genome oligonucleotide microarrays. The microarrays were scanned with a confocal LuxScanTM scanner, and the obtained images were then analyzed using the LuxScanTM 3.0 software (CapitalBio). For individual channel data extraction, faint spots for which the intensities were below 400 units after the background was subtracted in both channels (Cy3 and Cy5) were removed. A space- and intensity-dependent normalization based on LOWESS was employed. Significance Analysis of Microarrays (SAM, version 3.02) was performed to identify significantly differentially expressed genes.

### p65 DNA-binding Assay

U2OS cells were transiently transfected with DDRGK1 siRNAs or control siRNA. After 48 hours, nuclear extracts were prepared using a nuclear extract kit (Active Motif). The DNA-binding activity of p65 was determined using the NF-κB p65 transcription factor assay kit (Active Motif) according to the manufacturer’s instructions. 2.5 µg nuclear extracts of each sample were incubated with immobilized oligonucleotides containing the consensus κB site of 5′-GGGACTTTCC-3′ for 1 h. The p65 protein that was bound to κB DNA was then visualized by incubation with p65-specific antibody, HRP-conjugated secondary antibody and developing solution. The absorbance at 450 nm was measured with a multifunctional microplate reader.

### Luciferase Assay

Cells were plated in triplicates in 24-well plates the day before transfection. Reporter plasmids and DDRGK1 siRNAs or control siRNA were transiently transfected into cells together with the internal control plasmid pRL-TK. 24–48 h after transfection, firefly luciferase reporter activity was assayed using the Dual Luciferase Assay System (Promega, Madison, WI) according to the manufacturer’s instructions. Results were expressed as the mean firefly luciferase activity ±SD normalized by renilla luciferase activity from three independent experiments.

## Supporting Information

Table S1 The down-regulation of NF-κB target genes by DDRGK1 depletion in U2OS cells identified by microarray analysis. NF-κB target genes that were down-regulated by at least two-fold by both DDRGK1 siRNA1 and DDRGK1 siRNA2 were classified according to their biological functions. The gene ID designations were defined in accordance with the National Center for Biotechnology Information (NCBI) database. The ratios were calculated based on the value of Cy5 divided by the value of Cy3 for a given gene and are presented as the mean changes observed in the DDRGK1 siRNA1 and DDRGK1 siRNA2 transfection experiments.
(DOCX)
