# Supplementary material for: DDRGK1 Regulates NF-κB Activity by Modulating IκBα Stability
Source: PLoS One. 2013 May 10;8(5):e64231. doi: 10.1371/journal.pone.0064231 (PMC3651127; doi:10.1371/journal.pone.0064231)
Supplement: Table S1 — The down-regulation of NF-κB target genes by DDRGK1 depletion in U2OS cells identified by microarray analysis. NF-κB target genes that were down-regulated by at least two-fold by both DDRGK1 siRNA1 and DDRGK1 siRNA2 were classified according to their biological functions. The gene ID designations were defined in accordance with the National Center for Biotechnology Information (NCBI) database. The ratios were calculated based on the value of Cy5 divided by the value of Cy3 for a given gene and are presented as the mean changes observed in the DDRGK1 siRNA1 and DDRGK1 siRNA2 transfection experiments. (DOCX) [file pone.0064231.s001.docx]

| **Table S1. Down-regulation of NF-κB target genes in DDRGK1 depleted U2OS cells.** | | | |
| --- | --- | --- | --- |
| Gene ID | Official Symbol | Official full name | Ratio with control |
| **Cytokines/Chemokines and their modulators** | | |  |
| 6348 | CCL3 | Chemokine (C-C motif) ligand 3 | 0.43 |
| 6352 | CCL5 | Chemokine (C-C motif) ligand 5 | 0.37 |
| 6376 | CX3CL1 | Chemokine (C-X3-C motif) ligand 1 | 0.24 |
| 10148 | EBI3 | Epstein-Barr virus induced gene 3 | 0.42 |
| 3553 | IL1B | Interleukin 1, beta | 0.43 |
| 3586 | IL10 | Interleukin 10 | 0.40 |
| 3593 | IL12B | Interleukin 12B | 0.30 |
| 3458 | IFNG | Interferon gamma | 0.22 |
| **Transcription factors and their modulators** | | |  |
| 25909 | AHCTF1 | AT hook containing transcription factor 1 | 0.41 |
| 1044 | CDX1 | Caudal type homeobox 1 | 0.19 |
| 1758 | DMP1 | Dentin matrix acidic phosphoprotein 1 | 0.25 |
| 3660 | IRF2 | interferon regulatory factor 2 | 0.48 |
| 5241 | PGR | progesterone receptor | 0.28 |
| 6615 | SNAI1 | Snail homolog 1 | 0.41 |
| 6776 | STAT5A | Signal transducer and activator of transcription 5A | 0.48 |
| **Acute phase proteins** | | |  |
| 183 | AGT | Angiotensinogen (serpin peptidase inhibitor, clade A, member 8) | 0.37 |
| 722 | C4BPA | Complement component 4 binding protein, alpha | 0.45 |
| 3929 | LBP | Lipopolysaccharide binding protein | 0.44 |
| 6288 | SAA1 | serum amyloid A1 | 0.17 |
| 6289 | SAA2 | serum amyloid A2 | 0.16 |
| **Proteins involved in antigen presentation** | | |  |
| 239 | ALOX12 | Arachidonate 12-lipoxygenase | 0.18 |
| **Immunoreceptors** | |  |  |
| 1236 | CCR7 | Chemokine (C-C motif) receptor 7 | 0.29 |
| 941 | CD80 | CD80 molecule | 0.42 |
| 942 | CD86 | CD86 molecule | 0.30 |
| 3559 | IL2RA | Interleukin 2 receptor, alpha | 0.21 |
| 3579 | CXCR6 | Chemokine (C-X-C motif) receptor 6 | 0.38 |
| 3604 | TNFRSF9 | Tumor necrosis factor receptor superfamily, mumber 9 | 0.46 |
| 54210 | TREM1 | Triggering receptor expressed on myeloid cell 1 | 0.39 |
| **Cell-surface receptors** | | |  |
| 135 | ADORA2A | Adenosine A2a receptor | 0.24 |
| 2064 | ERBB2 | V-erb-b2 erythroblastic leukemia viral oncogene homolog 2 | 0.44 |
| 4988 | OPRM1 | Opioid receptor, mu 1 | 0.22 |
| 6337 | SCNN1A | Sodium channel, non-voltage-gated 1 alpha subunit | 0.29 |
| **Regulators of apoptosis** | | |  |
| 596 | BCL2 | B-cell CLL/lymphoma 2 | 0.44 |
| 8837 | CFLAR | CASP8 and FADD-like aoptosis regulator | 0.49 |
| 356 | FASLG | Fas ligand (TNF superfamily, member 6) | 0.22 |
| 5783 | PTPN13 | Protein tyrosine phosphatase, non-receptor type 13 | 0.20 |
| 29108 | PYCARD | PYD and CARD domain containing | 0.47 |
| **Growth factors, ligands, and their modulators** | | |  |
| 1440 | CSF3 | Colony stimulating factor 3 | 0.46 |
| 2056 | EPO | Erythropoietin | 0.33 |
| **Cell adhesion molecules** | | |  |
| 30835 | CD209 | CD209 molecule | 0.49 |
| 6403 | SELP | Seletin P (granule membrane protein 140kDa, antigen CD62) | 0.26 |
| **Enzymes** |  |  |  |
| 124 | ADH1A | Alcohol dehydrogenase 1A, alpha polypeptide | 0.33 |
| 417 | ART1 | ADP-ribosyltransferase 1 | 0.44 |
| 477 | ATP1A2 | ATPase, Na+/K+ transporting, alpha 2 polypeptide | 0.37 |
| 1806 | DPYD | Dihydropyrimidine dehydrogenase | 0.41 |
| 2797 | GNRH2 | Gonadotropin-releasing hormone 2 | 0.20 |
| 10855 | HPSE | Heparanase | 0.40 |
| 9388 | LIPG | Lipase, endothelial | 0.22 |
| 4318 | MMP9 | Matrix metallopeptidase 9 (gelatinase B, type IV collagenase) | 0.48 |
| 4842 | NOS1 | Nitric oxide synthase 1 | 0.47 |
| 4843 | NOS2 | Nitric oxide synthase 2 | 0.25 |
| 27035 | NOX1 | NADPH oxidase 1 | 0.35 |
| 5150 | PDE7A | Phosphodiesterase 7A | 0.43 |
| 7015 | TERT | Telomerase reverse transcriptase | 0.39 |
| 7051 | TGM1 | Transglutaminase 1 | 0.40 |
| **Stress response genes** | | |  |
| 1571 | CYP2E1 | Cytochrome P450, family 2, subfamily E, polypeptide 1 | 0.43 |
| 11184 | MAP4K1 | Mitogen-activated protein kinase kinase kinase kinase 1 | 0.37 |
| **Miscellaneous** | |  |  |
| 595 | CCND1 | Cyclin D1 | 0.47 |
| 9075 | CLDN2 | Claudin 2 | 0.46 |
| 1278 | COL1A2 | Collagen, type I, alpha 2 | 0.27 |
| 10964 | IFI44L | interferon-induced protein 44-like | 0.28 |
| 7849 | PAX8 | Paired box 8 | 0.23 |
| 5173 | PDYN | Prodynorphin | 0.43 |
| 7980 | TFPI2 | Tissue factor psthway inhibitor 2 | 0.31 |
